# MagGAT: Structure-Aware Spatiotemporal Graph Attention for Motor-Induced Magnetic Interference Compensation in Quadruped Robots

**DOI:** 10.3390/s26196048

**Published:** 2026-09-24

**Authors:** Qi Xue, You Li, Chen Wang, Qi Han

**Affiliations:** School of Cybersecurity, Faculty of Computing, Harbin Institute of Technology, Harbin 150001, China

**Keywords:** magnetometer, magnetic interference compensation, legged robot, graph attention network, spatiotemporal modeling, motor-induced interference

## Abstract

Permanent-magnet synchronous motors enable precise motion control in compact robots, but their leakage fields can corrupt onboard magnetometer measurements. Physics-based compensation is effective under fixed geometric conditions, whereas quadruped locomotion continuously changes the motor–sensor relationship. We propose MagGAT, a structure-aware spatiotemporal graph attention network that estimates compensated magnetic field signals from motor states and near-field measurements. A boom-mounted far-field magnetometer provides a pseudo-reference for supervised training and field-level evaluation. MagGAT combines a global–local dual-branch graph attention module for multi-motor spatial coupling with a gated recurrent unit for temporal disturbance modeling. Experiments on a Unitree Go2 quadruped used synchronized near-field and far-field measurements, joint angles, angular velocities, and commanded torques collected in indoor and outdoor environments. Across three runs with different random seeds, MagGAT achieved mean residual three-axis magnetic field RMSE values of 87.72–89.41 nT across the three test sequences and outperformed a physics-based fixed-parameter model, a static neural network method, and a temporal convolutional baseline. Ablation studies confirmed that both spatial graph modeling and temporal modeling contributed to compensation accuracy. These results support structure-aware spatiotemporal learning for suppressing motor-induced magnetometer interference on compact legged robots.

## 1. Introduction

Quadruped robots combine high mobility over uneven and discontinuous terrain with compact and highly integrated sensing and actuation [1,2]. These capabilities make them attractive for autonomous inspection, exploration, and emergency response in environments that are difficult for wheeled platforms [2,3]. Reliable localization and navigation are essential for such tasks because a legged robot must estimate its motion while its body undergoes repeated attitude and configuration changes [3,4]. Existing quadruped state estimation systems commonly fuse inertial measurements, joint encoders, cameras, or LiDAR to maintain navigation continuity [4,5].

Magnetic sensing can provide complementary information when satellite navigation is unavailable or unreliable. A magnetometer offers an absolute field reference for attitude and heading estimation, while spatial variations in the ambient magnetic field can support localization and mapping [6,7,8]. Magnetometers are also compact, passive, and suitable for continuous onboard measurements. These properties make reliable magnetic field measurements potentially valuable for legged robot navigation in GPS-denied environments. However, environmental magnetic features are useful only when platform-generated interference is sufficiently suppressed.

This requirement is difficult to satisfy on a compact quadruped robot. Multiple high-power motors, motor drivers, ferromagnetic structures, and high-current cables are located close to body-mounted sensors. Their fields can distort the ambient magnetic signal and degrade the measurement used by downstream heading or magnetic localization algorithms [9,10,11]. Permanent-magnet synchronous motors (PMSMs) are particularly important sources. Their rotor magnets, stator currents, and leakage fields produce disturbances that vary with motor angle, current, torque, and speed [12,13,14]. Classical platform-level compensation methods, including Tolles–Lawson-type models and their extensions, are effective in many aeromagnetic applications [15,16,17,18]. However, these methods generally assume that the sensor is separated from strong near-field sources or that the source–sensor geometry can be represented by stable parameters.

Learning-based magnetic anti-interference has also been studied from an anomaly detection and measurement recovery perspective. Su et al. proposed MagRobustNet, a UNet-like autoencoder framework that uses self-supervised masked reconstruction to identify interfered regions from reconstruction errors and recover their expected measurements from neighboring normal measurements [19]. The method is designed for magnetic tracking arrays affected by environmental hard/soft iron disturbances, nearby magnetic objects, and sensor saturation. In contrast, MagGAT does not first classify isolated measurements as anomalous or inpaint missing array regions, instead targeting continuously varying structure- and state-dependent interference from multiple nearby motors on a compact quadruped robot. Synchronized motor states and near-field magnetic measurements are processed through robot structure-aware global–local graph attention and temporal modeling in order to estimate a compensated three-axis field. In this way, MagRobustNet and MagGAT address complementary interference regimes, respectively, environmental anomaly recovery using spatial sensor redundancy and motor-induced dynamic compensation using actuator state and robot structure information.

Recent studies have identified several complementary requirements for magnetic measurements on motorized robotic platforms. Direction-aware calibration is needed because magnitude-only calibration may leave residual axis misalignment, scale-factor, and bias errors in multi-sensor magnetic systems [20]. At the source level, the spatial and temporal harmonics of PMSM magnetic fields and radial electromagnetic forces depend on rotor saliency, pole–slot configuration, winding design, and operating load; these harmonics can also excite mechanical vibration [21,22]. Physically structured analytical models and sensitivity analysis improve interpretability, but their accuracy remains tied to the modeled geometry and operating range [23,24]. For quadruped systems, whole-body motion coordination determines joint trajectories and actuator commands [25], while observer-based motor control shows that latent electrical states can be estimated from available drive signals [26]. Together, these findings motivate a compensation model that combines explicit motor state features with robot structure-aware and temporal learning while also retaining clear calibration and model validity boundaries.

Legged locomotion violates these assumptions because each joint motion changes the position and orientation of several motors relative to the body-mounted magnetometer. During walking, fixed geometry parameters cannot represent posture-dependent multi-motor coupling, so the measured disturbance becomes spatially coupled and temporally dynamic. Unlike our previous fixed-geometry physics-based model [14], MagGAT retains its motor component terms only as node features for learning posture-dependent multi-motor interference during locomotion.

We refer to this problem as dynamic spatiotemporal coupled magnetic interference (DST-CMI). Its magnitude and waveform vary with motor operating states, robot configuration, and time-varying motor–sensor geometry. DST-CMI is a descriptive formulation of the compensation problem rather than an independent physical model. Recovering a cleaner magnetic field signal is a prerequisite for evaluating magnetic heading and magnetic field-based localization on tightly integrated legged robots. Therefore, the present study focuses on field-level sensor compensation, and does not evaluate a complete navigation pipeline.

To address DST-CMI, we propose MagGAT, a structure-aware spatiotemporal graph attention network for magnetic interference compensation. A near-field magnetometer on the robot body provides the disturbed measurement. A far-field magnetometer mounted on a non-magnetic boom provides a pseudo-reference for supervised training and field-level evaluation. The reference is not treated as absolute navigation ground truth. MagGAT represents motors and magnetometers as graph nodes, uses global–local attention to model multi-motor spatial coupling, and applies recurrent modeling to capture temporal evolution. Figure 1 summarizes the training and inference workflow. The main contributions are as follows:

1. We formulate DST-CMI on compact legged robots as a structure-aware sensor compensation problem and propose MagGAT, a spatiotemporal graph attention model that combines global–local spatial attention with recurrent temporal modeling for magnetometer compensation.
2. We build a dual-magnetometer experimental platform on a Unitree Go2 quadruped robot and evaluate the method under indoor and outdoor conditions using field-level RMSE metrics.
3. We compare MagGAT with representative neural compensation baselines and conduct ablation studies to verify the contributions of the graph and temporal modules.

## 2. Materials and Methods

### 2.1. Motor-Induced Magnetic Interference

The actuator-level representation used in this study is adapted from our previously published physics-based PMSM interference model [14]. We do not repeat that model’s component derivation here. Instead, we retain only the measurable motor states required by MagGAT. The Unitree Go2 provides commanded torque, rotor-side angular velocity, and encoder angle for each of its twelve actuators. Commanded torque is used as an observable proxy for the torque-producing current, while the unknown proportionality is absorbed during network training. With the 6.33:1 reduction ratio, the transmission phase is defined as $q_{t}=\theta_{m}\left(t\right)/6.33$, with $\theta_{t}$ denoting the phase-aligned motor angle.

For each motor, these quantities are arranged as the following eight-dimensional node feature:

(1) $$\phi_{t}={\left[\tau_{\mathrm{cmd},t},\tau_{\mathrm{cmd},t}\cos\theta_{t},\tau_{\mathrm{cmd},t}\sin\theta_{t},\omega_{m,t},\cos\theta_{t},\sin\theta_{t},\cos q_{t},\sin q_{t}\right]}^{\;⊤}\in R^{8}.$$

The torque-dependent terms describe load-related excitation, the angle terms encode shaft-synchronous periodicity, and the $q_{t}$ terms describe transmission-synchronous modulation. This compact representation serves only as a physically motivated input to MagGAT. It is not presented as a new electromagnetic model or as a complete decomposition of motor harmonics. The graph and temporal modules learn the residual state-dependent coupling caused by joint motion and changing motor-to-sensor geometry.

### 2.2. MagGAT Network

The motor state representation in Section 2.1 describes each actuator independently, but the corresponding motor–sensor geometry changes during quadruped locomotion. As illustrated in Figure 2, each leg contains hip, thigh, and calf motors with relative positions and orientations that change as the joints rotate. For example, rotating the hip joint from its initial pose to positive or negative angles reconfigures the entire downstream leg and changes the spatial relationship between all three actuators and the body-mounted magnetometer. Therefore, motions of the four legs produce both local coupling among motors within one leg and global coupling between the twelve motors and the magnetometer. Because these geometric relationships evolve continuously, the observed interference is also temporally dependent.

MagGAT is designed to model this dynamic spatiotemporal coupling by combining spatial graph modeling with temporal sequence modeling. The network receives the near-field three-axis magnetic signal and synchronized motor state features from the twelve leg motors. It outputs a compensated three-axis magnetic signal that is trained to match the far-field reference magnetometer. The complete network architecture is shown in Figure 3.

#### 2.2.1. Spatial Graph Attention Module

The DST-CMI formulation suggests that the contribution of each motor to the measured magnetic disturbance is non-uniform and depends on both the robot structure and the current motor state. The implemented MagGAT configuration contains 15 graph nodes and produces a three-dimensional compensated magnetic field output. Twelve nodes represent the individual leg motors, while three magnetic component nodes encode the *x*-, *y*-, and *z*-axis components of the near-field measurement. The feature vector $\phi_{i,t}\in R^{8}$ defined in Equation (1) is assigned to motor node $v_{i}$, while the corresponding near-field component is assigned to each magnetic component node. Thus, the motor state input contains 96 physics-informed features per time step:

(2) $$\Phi_{t}={\left[\phi_{1,t}^{T},\ldots ,\phi_{12,t}^{T}\right]}^{T}\in R^{96}.$$

Because the motor and magnetic component nodes have different physical meanings, separate learnable projections map them into a common latent space:

(3) $$h_{i,t}^{\left(0\right)}=\left\{\begin{array}{cc} W_{m}\phi_{i,t}+b_{m},\; & i=1,\ldots ,12,\\ w_{B,a}B_{\mathrm{near},t}^{\left(a\right)}+b_{B,a},\; & i=v_{B,a},\;a\in \left\{x,y,z\right\}. \end{array}\right.$$

This node-wise construction retains the identity of every actuator and magnetic field component instead of treating all inputs as a single unstructured vector.

Graph attention [27] is then used to learn state-dependent influence weights over the structural neighborhood of each node. Each spatial branch contains three stacked GAT layers, with four attention heads and 512-dimensional hidden features per layer. For branch b∈{loc,glo} and attention head *k*, the unnormalized compatibility score and normalized attention coefficient between nodes *i* and *j* are

(4) $$\begin{array}{cc} e_{ij,t}^{\left(b,k\right)} & =\mathrm{LeakyReLU}\left[a_{b,k}^{T}\left(W_{b,k}h_{i,t}^{\left(0\right)}∥W_{b,k}h_{j,t}^{\left(0\right)}\right)\right], \end{array}$$

(5) $$\begin{array}{cc} \alpha_{ij,t}^{\left(b,k\right)} & =\frac{\exp\;\left(e_{ij,t}^{\left(b,k\right)}\right)}{\sum_{r\in N_{i}^{\left(b\right)}}\exp\;\left(e_{ir,t}^{\left(b,k\right)}\right)},\;j\in N_{i}^{\left(b\right)}, \end{array}$$

where $N_{i}^{\left(b\right)}$ is determined by the topology of branch *b* and ‖ denotes concatenation. The updated node representation is obtained by aggregating the neighboring features over *K* attention heads:

(6) $$h_{i,t}^{\left(b\right)}=\overset{K}{\underset{k=1}{∥}}\sigma \left(\sum_{j\in N_{i}^{\left(b\right)}}\alpha_{ij,t}^{\left(b,k\right)}W_{b,k}h_{j,t}^{\left(0\right)}\right).$$

Consequently, the spatial contribution of a motor is conditioned jointly on its current operating state and its structural neighbors. The learned coefficients are used as data-driven feature weights, not exact magnetic coupling coefficients.

#### 2.2.2. Global–Local Dual-Branch Fusion

The two graph branches encode complementary spatial relations. The local edge set connects the hip, thigh, and calf motor nodes within each leg, preserving the short-range mechanical structure and the coupling induced by downstream joint motion. The global edge set forms a star-shaped topology between all twelve motor nodes and the magnetometer node, representing the superposed platform-level influence of the four legs on the body-mounted sensor. The two branches use independent attention parameters because an important neighbor within one leg need not have the same role in the global motor–sensor relation.

Let $s_{t}^{\mathrm{loc}}$ and $s_{t}^{\mathrm{glo}}$ denote the branch-level representations obtained by collecting the corresponding node embeddings and projecting them to the same feature dimension. A learnable gate combines the two representations:

(7) $$\begin{array}{cc} g_{t} & =\mathrm{sigmoid}\left(W_{g}\left[s_{t}^{\mathrm{loc}}∥s_{t}^{\mathrm{glo}}\right]+b_{g}\right), \end{array}$$

(8) $$\begin{array}{cc} s_{t} & =g_{t}⊙s_{t}^{\mathrm{glo}}+\left(1-g_{t}\right)⊙s_{t}^{\mathrm{loc}}, \end{array}$$

where ⊙ denotes element-wise multiplication. The gate is evaluated at every time step, allowing the network to vary the balance between global platform-level coupling and local same-leg coupling as the robot changes posture. This fusion avoids imposing a fixed contribution from either topology, while the global-only and local-only ablations in Section 3.2 isolate the roles of each branch.

#### 2.2.3. Temporal Modeling

The fused vector $s_{t}$ describes the spatial coupling at one instant, but does not by itself represent how the interference evolves during continuous motion. Therefore, MagGAT forms the following causal sequence:

(9) $$S_{t}=\left[s_{t-L+1},\ldots ,s_{t}\right],\;L=20.$$

Both magnetic and motor signals are sampled at 10 Hz, so the sequence covers the current sample and the preceding 1.9 s of history. Only present and past samples are used, which permits causal compensation.

The temporal module stacks three gated recurrent unit (GRU) layers. Following the GRU formulation introduced by Cho et al. [28], each layer updates its hidden state for sequence element $s_{\ell }$ according to

(10) $$\begin{array}{cc} r_{\ell } & =\mathrm{sigmoid}\left(W_{r}s_{\ell }+U_{r}h_{\ell -1}+b_{r}\right), \end{array}$$

(11) $$\begin{array}{cc} u_{\ell } & =\mathrm{sigmoid}\left(W_{u}s_{\ell }+U_{u}h_{\ell -1}+b_{u}\right), \end{array}$$

(12) $$\begin{array}{cc} {\tilde{h}}_{\ell } & =\tanh\left(W_{h}s_{\ell }+U_{h}\left(r_{\ell }⊙h_{\ell -1}\right)+b_{h}\right), \end{array}$$

(13) $$\begin{array}{cc} h_{\ell } & =u_{\ell }⊙h_{\ell -1}+\left(1-u_{\ell }\right)⊙{\tilde{h}}_{\ell }. \end{array}$$

The reset gate $r_{\ell }$ controls how much previous information contributes to the candidate state, whereas the update gate $u_{\ell }$ controls the persistence of the hidden state. These operations allow the model to retain recent motor and magnetic history while adapting to changes in gait phase and posture.

The final hidden state is mapped by a multilayer perceptron with one 1024-dimensional hidden layer and a three-dimensional linear output to obtain the compensated magnetic field

(14) $${\hat{B}}_{g,t}=f_{\mathrm{MLP}}\;\left(h_{t}\right)\in R^{3}.$$

The network is trained against the synchronized far-field pseudo-reference by minimizing the mean squared error

(15) $$L_{\mathrm{MSE}}=\frac{1}{3N}\sum_{t=1}^{N}{∥{\hat{B}}_{g,t}-B_{\mathrm{far},t}∥}_{2}^{2}.$$

The far-field measurement is required only as a supervised target during training and evaluation. During inference, MagGAT uses the near-field magnetometer and the motor state features to produce the compensated magnetic field estimate.

### 2.3. Experimental Platform

Experiments were conducted on a Unitree Go2 quadruped robot equipped with two three-axis magnetometers, as shown in Figure 4. An RM3100 magnetometer manufactured by PNI Sensor Corporation, Santa Rosa, CA, USA, was mounted on the robot body as the near-field sensor and was directly exposed to motor-induced interference. An HSF200 magnetometer manufactured by Xi’an Huashun Measurement and Control Technology Co., Ltd., Xi’an, China, was mounted at the tip of a non-magnetic carbon fiber boom as the far-field sensor. The far-field sensor was used as a pseudo-reference for supervised learning and field-level evaluation.

The boom places the reference magnetometer approximately 60 cm away from the robot body, motors, motor drivers, and high-current wiring; therefore, the reference measurement is expected to be much less affected by platform-induced magnetic interference than the near-field body measurement. In this paper, this far-field measurement is used only as a practical reference for magnetic field compensation.

Before data acquisition, both magnetometers were calibrated at the same fixed location using multi-orientation ellipsoid fitting and were mechanically aligned to the same nominal robot-fixed axes. For sensor j∈{near,far}, the calibrated magnetic flux density vector is computed as

(16) $$B_{j,t}^{\mathrm{cal}}=s_{j}C_{j}\left(B_{j,t}^{\mathrm{raw}}-b_{j}\right),$$

where $b_{j}$ is the hard iron bias, $C_{j}$ corrects scale, non-orthogonality, and soft iron distortion, and $s_{j}$ maps both outputs to a common nanotesla scale. The same calibration and fixed axis mapping were applied to all sequences without sequence-specific fitting. This procedure reduces systematic sensor differences but cannot eliminate residual misalignment, calibration uncertainty, spatial gradients, or boom motion [20]. The boom measurement is treated as a practical pseudo-reference, and RMSE denotes agreement with this calibrated sensor rather than error relative to the unknown absolute ambient field.

### 2.4. Validation of the Boom-Mounted Pseudo-Reference

To examine whether the boom-mounted magnetometer can provide a stable field-level reference, an additional validation experiment was conducted with the robot body fixed in a stationary configuration. As shown in Figure 5, the near-field magnetometer was mounted on the robot body, while the far-field magnetometer was mounted at the tip of the non-magnetic boom. During this test, the ambient magnetic environment and the robot pose remained approximately unchanged, and only selected leg motors were actuated to induce platform-generated magnetic interference. This setting isolates the effect of motor actuation from whole-body motion, so the measured magnetic field variation mainly reflects motor-induced interference rather than changes in the external geomagnetic field or the overall robot attitude.

Figure 6 compares the magnetic field variations measured by the body-mounted RM3100 near-field magnetometer and the boom-mounted HSF200 far-field magnetometer when different leg motors were actuated separately while keeping the robot body fixed. Both signals were processed using a 0.2–1.5 Hz band-pass filter in order to isolate fluctuations related to motor motion. The standard deviation of the filtered near-field signal was 414.4952 nT and that of the filtered far-field signal was 10.8381 nT, yielding a near-to-far standard deviation ratio of 38.24. This quantitative difference indicates that the boom-mounted sensor is substantially less affected by platform-induced motor interference than the body-mounted near-field sensor under the tested conditions. Therefore, the magnetic field measured by the boom-mounted magnetometer is used as the reference value for supervised training and field-level evaluation in this paper.

### 2.5. Coupled Multi-Motor Motion Test

To characterize the temporal association between controlled motor actuation and the onboard magnetic measurement, low-level commands were used to actuate selected motors while keeping the robot body fixed. Figure 7 compares the joint angle trajectories with the magnetic field variation measured by the body-mounted magnetometer.

### 2.6. Training and Evaluation Setup

Data were collected in the outdoor and indoor environments shown in Figure 8. The outdoor site was an open concrete area, while the indoor site was a corridor-like area of approximately 200 m^2^. During acquisition, the near-field and far-field magnetometers were recorded synchronously with the motor and kinematic states. One outdoor sequence was partitioned into training and validation subsets, with the validation subset used for early stopping. Generalization performance was subsequently evaluated on three independent sequences collected on different days from the training sequence, comprising one outdoor sequence and two indoor sequences. These independent test sequences are denoted RMSE1, RMSE2, and RMSE3.

The source outdoor recording used for model development lasted approximately 31 min, corresponding to approximately 18,600 synchronized samples at the nominal 10-Hz sampling rate. A continuous 90% portion (approximately 27.9 min and 16,740 samples) was assigned to training and the remaining non-overlapping 10% portion (approximately 3.1 min and 1860 samples) for validation and early stopping. Each of the three independent test sequences lasted approximately 10–15 min, corresponding to approximately 6000–9000 samples per sequence at 10 Hz, and was acquired on a different day from the training sequence.

The continuous recordings were divided chronologically into mutually exclusive temporal segments prior to sliding window generation. The 20-sample causal windows were then constructed independently within each subset. Windows that would cross a subset boundary were discarded, and no window was randomly distributed or reassigned across the training, validation, and test subsets. Consequently, adjacent windows may overlap only within the same subset, with no temporal overlap between model development and test data. At the 10-Hz sampling rate, each window contains the current sample and the preceding 19 samples, corresponding to 1.9 s of history and a 2.0-s sample span. The same temporal split and window generation procedure were used for every compared method.

The evaluation metric is the residual RMSE between the compensated magnetic field and the far-field reference over all three axes:

(17) $$\mathrm{RMSE}=\sqrt{\frac{1}{3T}\sum_{t=1}^{T}\sum_{i\in \{x,y,z\}}{\left(B_{g,i}\left(t\right)-B_{\mathrm{ref},i}\left(t\right)\right)}^{2}}$$

where $B_{g}$ is the compensated magnetic field output and $B_{\mathrm{ref}}$ is the far-field reference. After ellipsoid compensation and common-scale calibration, the corrected magnetic measurements were divided by a constant factor of 30,000 for numerical conditioning before being fed into the network and were restored to nanotesla units for evaluation. Training used the Adam optimizer with a learning rate of $10^{-5}$, a batch size of 512, and early stopping with a patience of 30 epochs. Training was performed on an NVIDIA RTX 4090 GPU with CUDA 11.2.

## 3. Results

### 3.1. Comparison with Baseline Methods

This section compares MagGAT with three representative compensation baselines. Physics-RLS denotes the physics-based PMSM compensation method from our previous work [14], in which the interference coefficients are identified using recursive least squares and then kept fixed during the present test sequences. The second baseline is the fully connected neural network calibration method of Laoué et al. [29], which performs static regression of magnetic interference. The third is MagTCN [30], a temporal convolutional network that models dynamic magnetic interference over time. All methods were evaluated using the same data split and the same residual RMSE metric.

As shown in Table 1, MagGAT achieves the lowest RMSE on all three test sequences. Physics-RLS obtains RMSE values of 126.26, 123.69, and 121.28 nT, respectively, outperforming the other two baselines on all three test sequences but remaining consistently less accurate than MagGAT. This result indicates that the physics-based features capture meaningful motor interference characteristics, whereas a single fixed parameter set identified under a limited calibration configuration is insufficient for quadruped locomotion. As the joints move, the relative positions and orientations of the twelve motors and the body-mounted magnetometer vary continuously, causing the effective coupling coefficients to change with posture and gait phase. In this way, the lower residual errors of MagGAT support the use of state-dependent spatial attention and temporal modeling rather than fixed compensation parameters for the tested dynamic robot motions. Figure 9 shows representative indoor and outdoor residual time series for all compared methods.

**Table 1:** Comparison of residual magnetic field RMSE with baseline methods. MagGAT is reported as the mean ± standard deviation over three runs with random seeds 40, 41, and 42, consistent with Table 2. Bold values indicate the best performance in each test sequence. Lower is better.

Method	RMSE1 (nT)	RMSE2 (nT)	RMSE3 (nT)
MagGAT (three runs)	**88.60 ± 0.62**	**89.41 ± 0.46**	**87.72 ± 0.55**
Physics-RLS [14]	126.26	123.69	121.28
MagTCN [30]	141.02	132.63	140.60
Laoué et al. [29]	144.77	145.12	145.93

**Figure 9:**
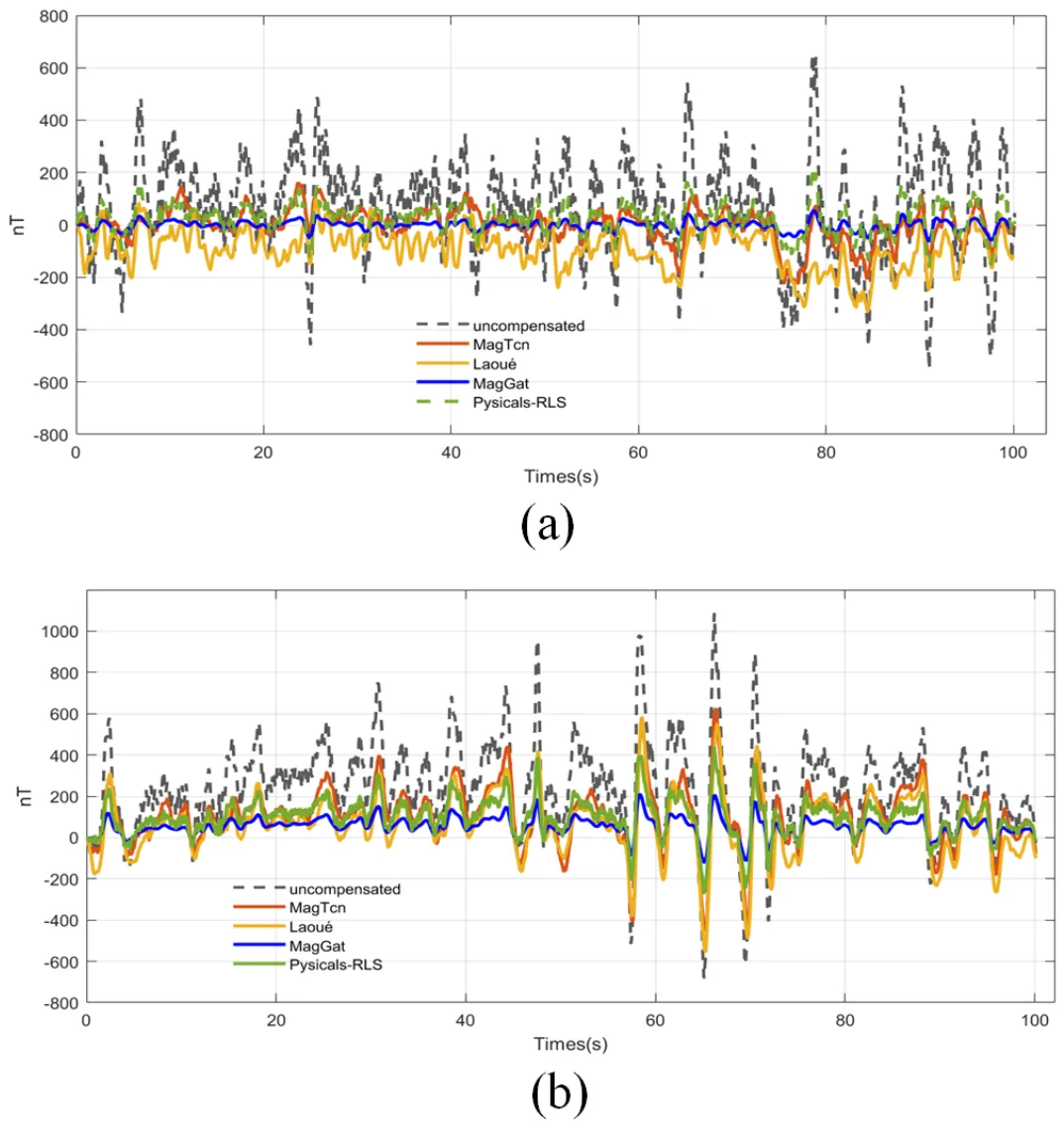
Time series comparison of magnetic field residuals over representative 100-s segments from (**a**) an outdoor test sequence and (**b**) an indoor test sequence. The residuals are computed relative to the far-field reference magnetometer. Physics-RLS reduces the uncompensated interference, whereas MagGAT maintains the smallest residual fluctuations during dynamic robot motion.

**Table 2:** Residual RMSE comparison across ablation variants. MagGAT is reported as the mean ± standard deviation over three runs with different random seeds; the ablation variants are single-run results. Bold values indicate the best performance in each test sequence. Lower is better.

Method	RMSE1 (nT)	RMSE2 (nT)	RMSE3 (nT)
MagGAT (three runs)	**88.60 ± 0.62**	**89.41 ± 0.46**	**87.72 ± 0.55**
Global-branch only	108.64	100.96	97.27
Local-branch only	123.70	116.26	120.36
Flattened-feature GRU	187.08	205.54	176.04
Double-branch GAT only	153.50	179.44	149.53

### 3.2. Ablation Studies

To evaluate the contributions of individual model components, ablation studies were conducted using the same data split, sequence length, sampling rate, optimizer, and batch size as the full MagGAT model. The compared variants include global branch only, local branch only, GRU only, and double-branch GAT without explicit temporal modeling. For a parameter-matched comparison, both the number of attention heads and the hidden feature dimension of the single-branch global-only and local-only variants were correspondingly increased so that the trainable parameter count of each variant matched that of the full MagGAT model. This design reduces the possibility of the observed performance differences being caused merely by a smaller model capacity, and instead focuses the ablation on the effect of global–local structural modeling.

Table 2 shows that removing either the global–local fusion or the temporal module increases the residual RMSE. The flattened-feature GRU and double-branch GAT-only variants also remain less accurate than MagGAT, supporting the joint use of robot structure-aware spatial modeling and temporal history under the tested conditions.

## 4. Discussion

The results support the main hypothesis that motor-induced magnetometer interference on compact legged robots is both structure-dependent and time-dependent. The global–local graph branches allow the model to represent different spatial scales of interference, while the recurrent module captures recent history in the motor and magnetic measurements. This combination is consistent with the physical observation that motor leakage fields depend on motor operating state, sensor source geometry, and robot motion.

The coupling pathway can be interpreted as follows. A gait-level command is converted by the whole-body controller into coordinated joint trajectories and actuator commands, subject to contact and kinematic constraints [25]. These commands determine the measured joint angles, angular velocities, and commanded torques used by MagGAT, while the resulting leg motion continuously changes the relative positions and orientations of the motors, cables, and body-mounted magnetometer. In parallel, motor electromagnetic harmonics can excite structural vibration [21,22], which may further modulate the measured field through small sensor pose changes and cable motion. Thus, the observed synchronization between actuator motion and magnetic variation supports a state-dependent association, but does not prove that a single electromagnetic mechanism is solely responsible for every measured fluctuation.

The present model uses the motor states available from the Unitree interface, namely, the angle, angular velocity, and commanded torque. Direct phase current measurements, reconstructed *d*–*q* currents, inverter switching states, bus voltage, or observer-estimated flux and rotor states could provide additional information about the instantaneous electromagnetic operating condition. For example, hybrid active-flux observation has been used to recover rotor state information for PMSM drives under difficult low-speed conditions [26]. Incorporating such electrical states may improve separation of current-driven leakage fields from geometry- and vibration-related effects, but these signals were not available in the present dataset.

Magnetic coupling is also sensitive to geometry and operating conditions. Integrated magnetic network modeling with pointwise sensitivity analysis has demonstrated how geometric variables can alter coupled magnetic responses and optimization directions [24]. While MagGAT addresses this variability through state-conditioned attention, the current experiments do not constitute a formal sensitivity analysis over sensor placement, payload, motor temperature, gait, and speed; therefore, the reported robustness is only applicable within the tested configurations. A controlled geometric and operational sensitivity study is an important next step for assessing transfer to other sensor layouts and quadruped platforms.

This study focuses on field-level magnetometer compensation rather than navigation-level heading evaluation. The far-field magnetometer provides a practical pseudo-reference for supervised training and residual-field assessment, but does not constitute an independent-heading ground truth. The present results show that the near-field measurement can be compensated toward the far-field reference under the tested indoor and outdoor conditions, but do not establish absolute heading accuracy. Full navigation-level validation requires an independent heading reference, such as motion capture or a high-precision external vision system.

The current study has several other limitations. First, the far-field boom reference is a practical pseudo-reference rather than an absolute disturbance-free measurement. Second, the training and testing data were collected on one robot platform, so cross-platform transfer remains to be evaluated. Third, further experiments with different gaits, speeds, and payload configurations could provide a more complete picture of generalization.

## 5. Conclusions

This paper presents MagGAT, a structure-aware spatiotemporal graph attention network for compensating motor-induced magnetometer interference on compact legged robots. The method uses motor angles, angular velocities, commanded torques, and near-field magnetic measurements to estimate a compensated magnetic field with respect to a far-field boom-mounted reference magnetometer. Across three runs with different random seeds, experiments on a Unitree Go2 quadruped robot yielded mean residual three-axis magnetic field RMSE values of 87.72–89.41 nT across the three test sequences. MagGAT outperformed the tested physics-based fixed-parameter, static neural network, and temporal convolutional baselines. Ablation studies confirmed that both the graph-based spatial module and the GRU-based temporal module improved compensation performance. Future work will validate the compensated magnetic signal in a full navigation pipeline using an independent heading ground truth, and will additionally investigate lightweight deployment and cross-platform generalization.

## Figures and Tables

**Figure 1:**
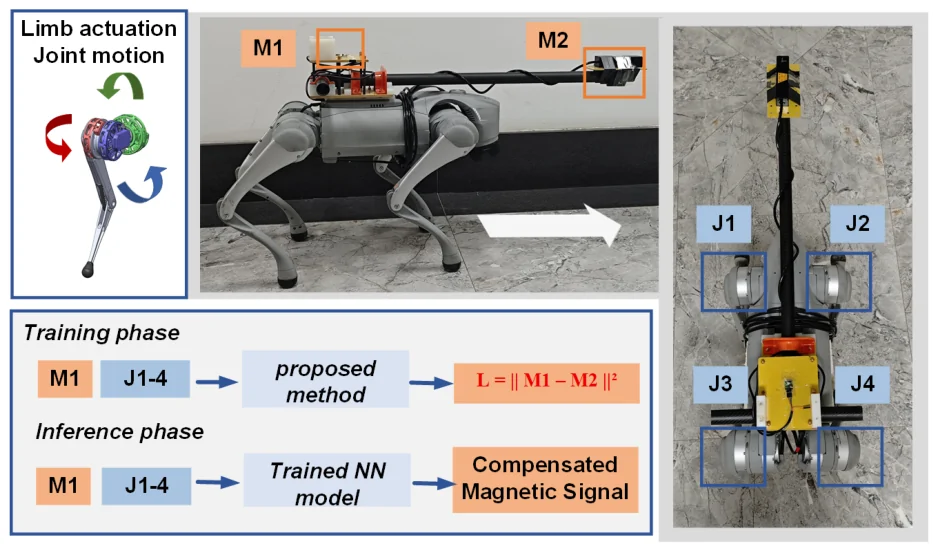
Overview of the proposed magnetic interference compensation framework. Motor motion and motor currents induce magnetic disturbances on the near-field magnetometer. During training, the network learns a supervised mapping from near-field magnetic measurements and joint motor states to the far-field reference magnetic signal. During inference, only the near-field magnetometer and motor data are used to generate the compensated magnetic signal.

**Figure 2:**
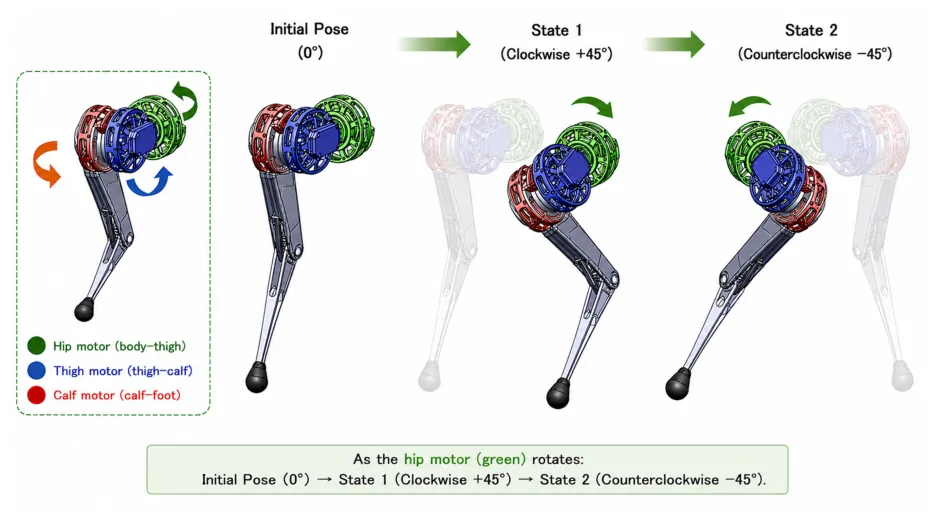
Posture-dependent motor geometry in one quadruped leg. Rotation of the hip joint from the initial pose to positive and negative angles changes the positions and orientations of the hip, thigh, and calf motors relative to the body-mounted magnetometer. Similar joint motion across all four legs produces time-varying local and global magnetic interference coupling.

**Figure 3:**
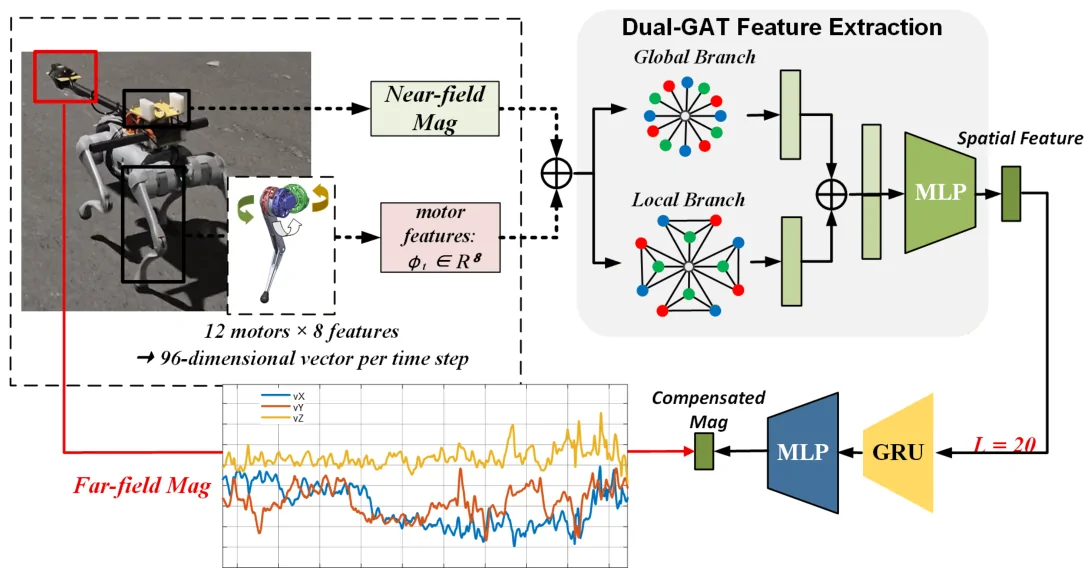
Overall architecture of MagGAT. A dual-branch graph attention network extracts spatial features from motor and magnetometer nodes. The fused spatial features are then fed into a GRU-based temporal module, followed by a multilayer perceptron that outputs the compensated magnetic field.

**Figure 4:**
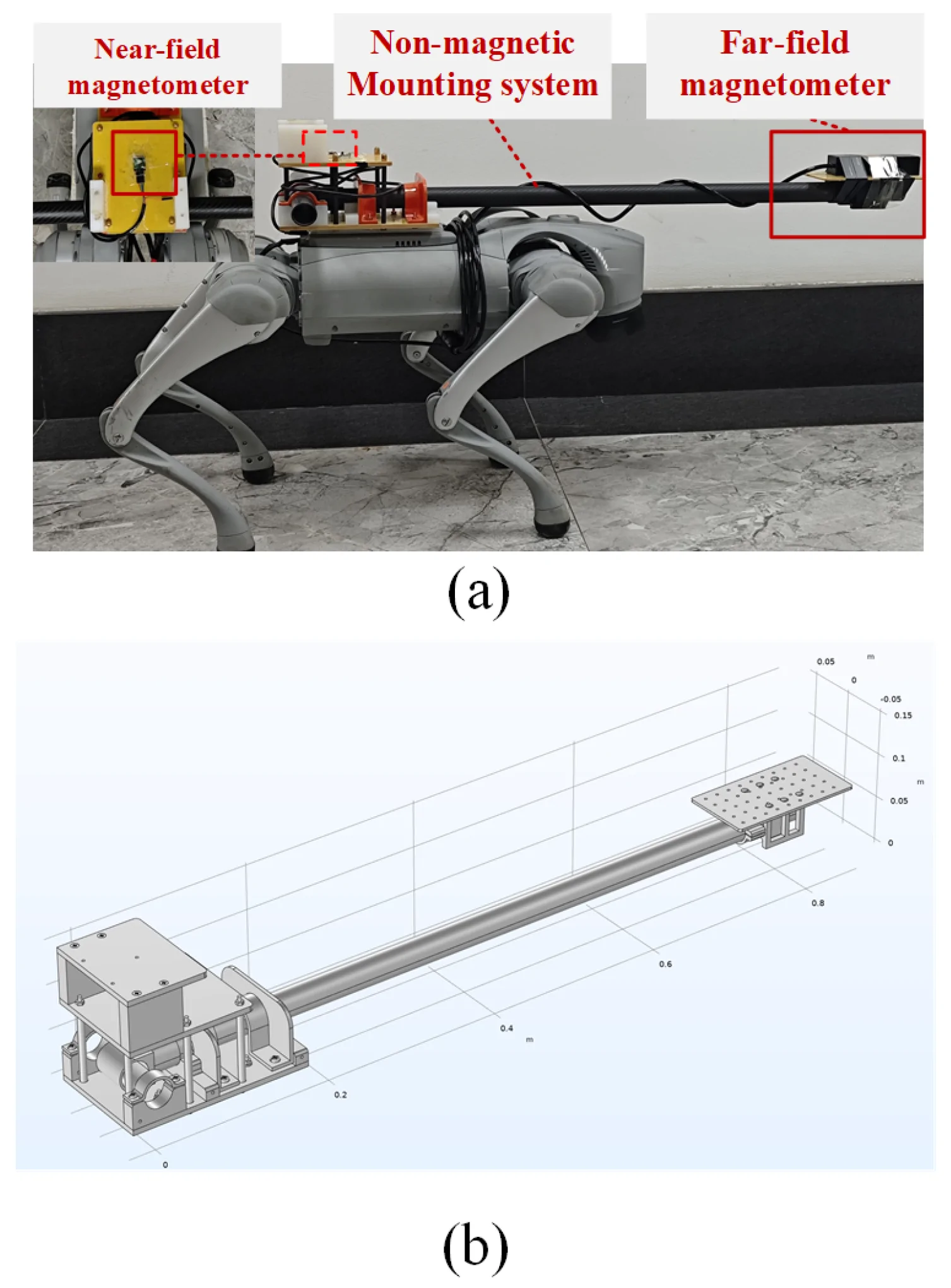
Experimental platform. (**a**) Unitree Go2 quadruped equipped with a body-mounted RM3100 near-field magnetometer and an HSF200 far-field reference magnetometer; (**b**) CAD representation of the non-magnetic boom. The far-field sensor is mounted on a boom made from carbon fiber and fiberglass with non-ferromagnetic fasteners.

**Figure 5:**
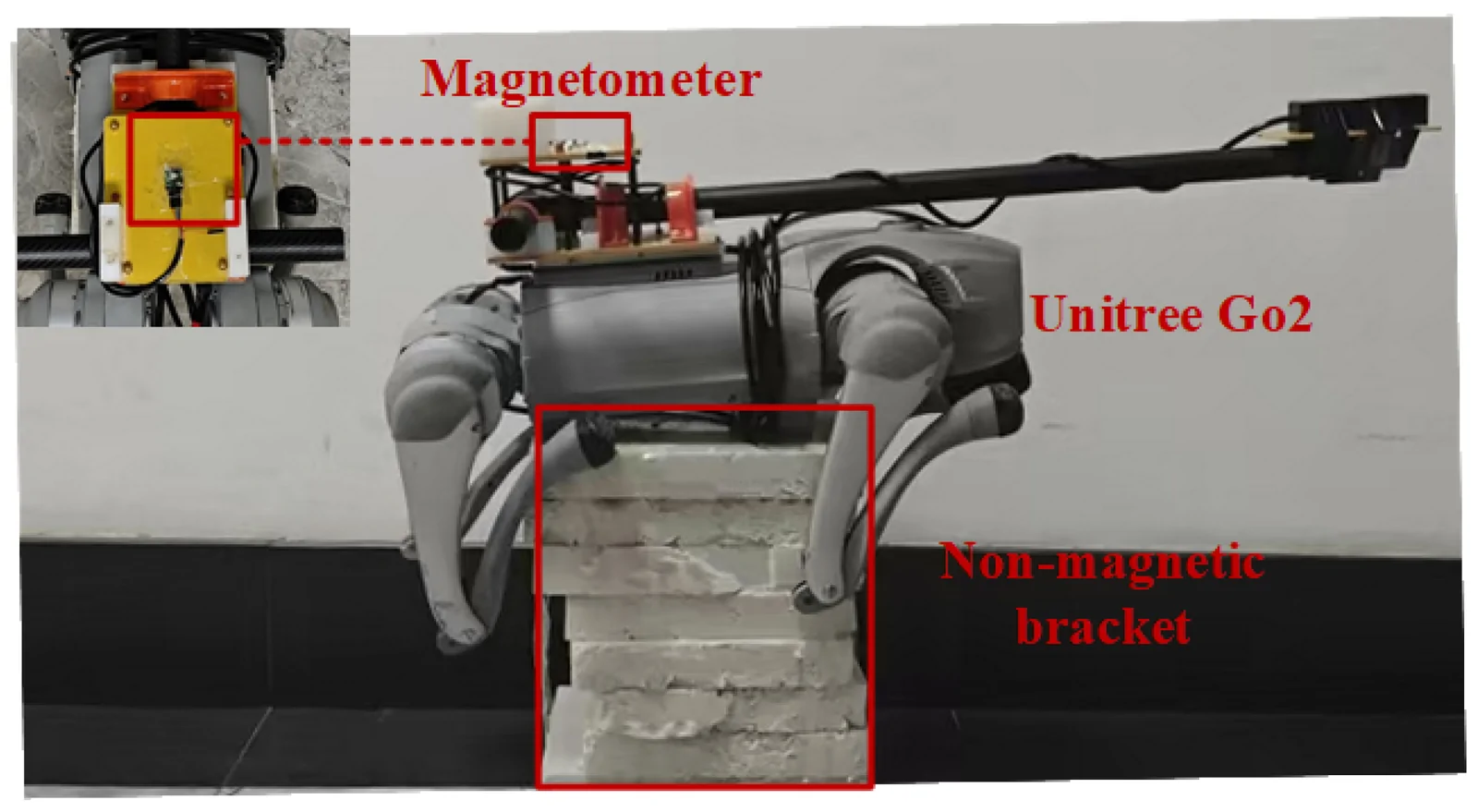
Experimental setup for validating the boom-mounted pseudo-reference. The robot body was fixed while selected leg motors were actuated to induce platform magnetic interference. The near-field magnetometer was mounted on the robot body and the far-field magnetometer was mounted at the tip of the non-magnetic boom.

**Figure 6:**
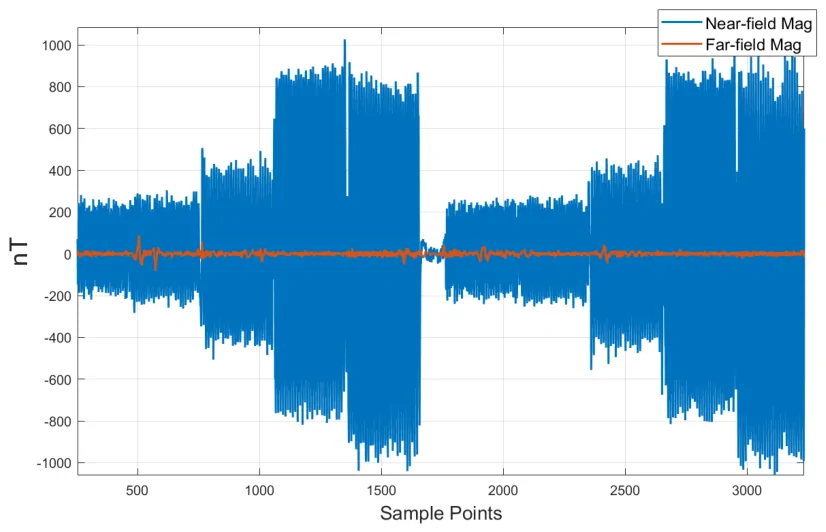
Comparison of magnetic field variations measured by the body-mounted RM3100 near-field magnetometer and the boom-mounted HSF200 far-field magnetometer after 0.2–1.5 Hz band-pass filtering. With the robot body fixed and different leg motors actuated separately, the standard deviations of the near-field and far-field signals were 414.4952 nT and 10.8381 nT, respectively. The overlap between traces does not affect the scientific interpretation because the comparison focuses on their relative fluctuation amplitudes.

**Figure 7:**
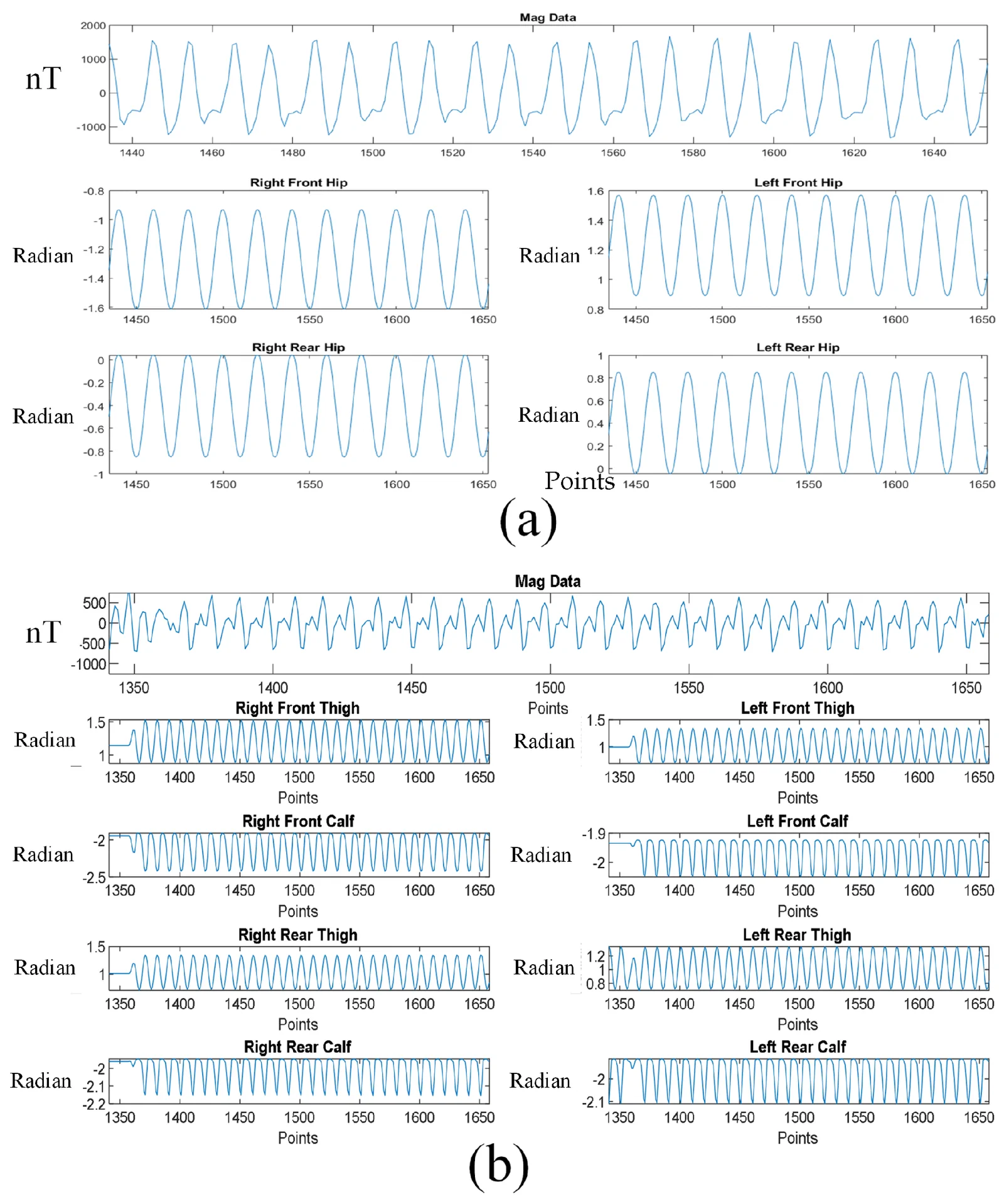
Magnetic field variation measured by the near-field magnetometer and the synchronized joint-angle trajectories during controlled motor actuation: (**a**) hip joint actuation and (**b**) thigh and calf joint actuation. The horizontal coordinate denotes the sample index (sampling rate: 10 Hz), while the vertical coordinates are magnetic field magnitude (nT) and joint angle (rad), respectively. The synchronized variations indicate a temporal association between joint motion and the onboard magnetic measurement.

**Figure 8:**
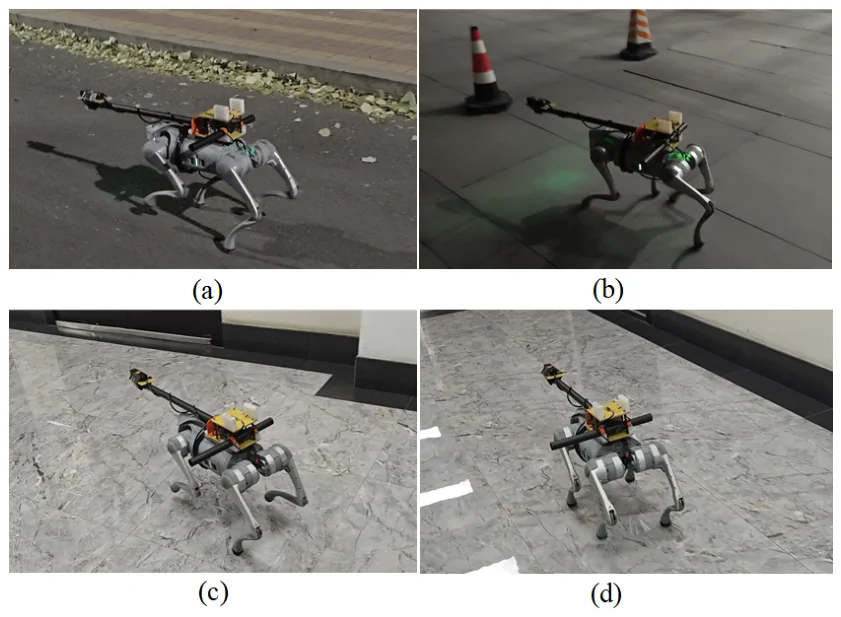
Experimental environments. Subfigures (**a**,**b**) show outdoor data collection scenes, while subfigures (**c**,**d**) show indoor scenes.

## Data Availability

Data are available from the corresponding author upon reasonable request.

## Abbreviations

The following abbreviations are used in this manuscript:

GAT	Graph Attention Network
GRU	Gated Recurrent Unit
FOC	Field-Oriented Control
DST-CMI	Dynamic Spatio-Temporal Coupled Magnetic Interference
IMU	Inertial Measurement Unit
MLP	Multi-Layer Perceptron
PMSM	Permanent-Magnet Synchronous Motor
RMSE	Root Mean Square Error
